# Supplementary material for: Cryo-EM structure of the hibernating Thermus thermophilus 100S ribosome reveals a protein-mediated dimerization mechanism
Source: Nat Commun. 2018 Oct 9;9:4179. doi: 10.1038/s41467-018-06724-x (PMC6177447; doi:10.1038/s41467-018-06724-x)
Supplement: Supplementary file 1 — Supplementary Information [file 41467_2018_6724_MOESM1_ESM.pdf]

Cryo-EM structure of the hibernating *Thermus thermophilus* 100S ribosome reveals a protein-mediated dimerization mechanism

R. K. Flygaard *et al.* (2018)

Supplementary Information

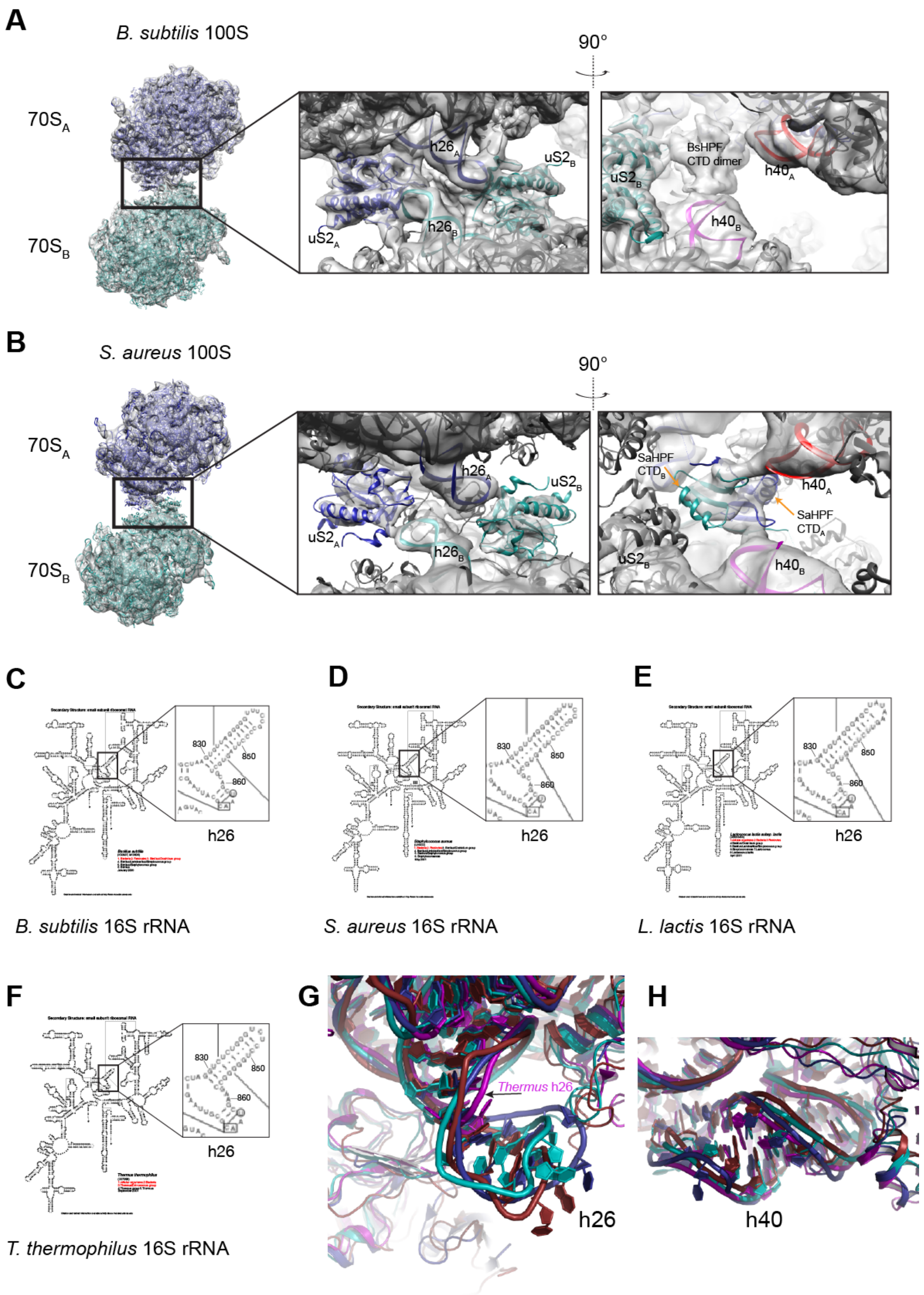
A
B. subtilis 100S
90°
70SA
70SB
h26A
uS2B
uS2A
h26B
BsHPF
CTD dimer
uS2B
h40A
h40B
B
S. aureus 100S
90°
70SA
70SB
h26A
uS2B
uS2A
h26B
SaHPF
CTDB
h40A
SaHPF
CTDA
uS2B
h40B
C
830
850
860
h26
B. subtilis 16S rRNA
D
830
850
860
h26
S. aureus 16S rRNA
E
830
850
860
h26
L. lactis 16S rRNA
F
830
850
860
h26
T. thermophilus 16S rRNA
G
Thermus h26
h26
H
h40

Supplementary Figure 1

Stabilization of ribosome dimerization interface in *B. subtilis* and *S. aureus* and comparison to *T. thermophilus* ribosome. **A)** *B. subtilis* 100S ribosome cryo-EM density map (EMD-3664) fitted with model (PDB entry 5NJT [10.2210/pdb5NJT/pdb]) and **B)** *S. aureus* 100S ribosome cryo-EM density map (EMD-3637) fitted with model (PDB entry 6FXC [10.2210/pdb6FXC/pdb]). In A) and B) 70S ribosomes comprising dimers are denoted A and B respectively. Close-up view on the left shows 16S rRNA h26 from one ribosome copy interacting with ribosomal protein uS2 on the other ribosome copy. Close-up view on the right shows proximity of 16S rRNA h40 to CTD homodimer of HPF enabling interactions. **C-F)** 16S rRNA secondary structure diagrams of C) *B. subtilis*, D) *S. aureus*, E) *L. lactis* and E) *T. thermophilus* (all diagrams from http://www.rna.icmb.utexas.edu/). Close-up views all show h26 region of 16S rRNA. Numbering of rRNAs is according to *E. coli*. **G)** Superposition of ribosome structures focused on 16S rRNA h26 clearly showing the shorter length of h26 in *T. thermophilus* (purple, PDB 4V8H [10.2210/pdb4V8H/pdb]) compared to *B. subtilis* (teal, PDB 5NJT), *L. lactis* (ruby, PDB 5MYJ [10.2210/pdb5MYJ/pdb]) and *S. aureus* (blue, PDB 5NGM [10.2210/pdb5NGM/pdb]). **H)** Structures and colors are as in G) but now viewing 16S rRNA h40 showing no difference in length between *T. thermophilus* ribosome compared to the other three.

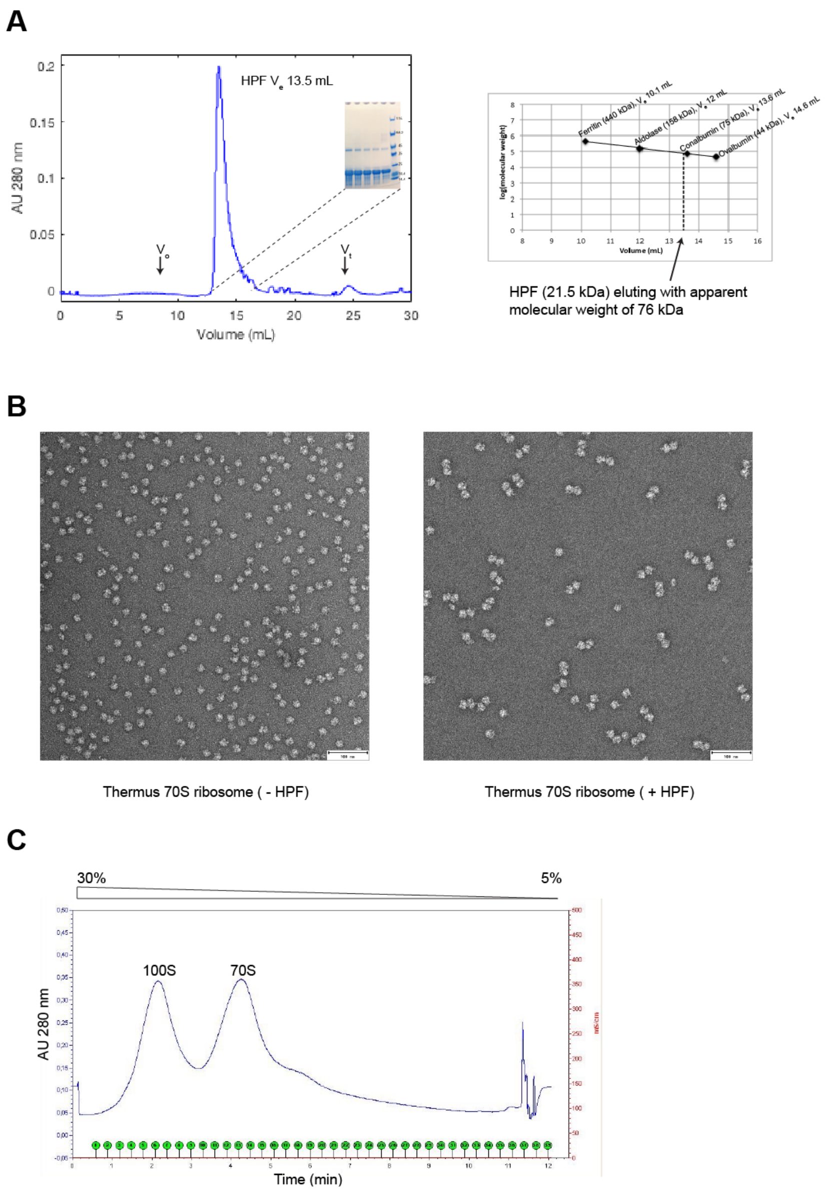

Supplementary Figure 2

In vitro characterization of purified *Tt*HPF and formation of 100S ribosomes. **A)** Size exclusion gel filtration chromatogram of *Tt*HPF from Superdex200 Increase 10/300. Purified *Tt*HPF protein elutes in a single peak with an elution volume corresponding to a globular protein of molecular weight 76 kDa when compared to standard calibration of the column (right figure). Gel insert shows high purity of *Tt*HPF protein. Band migrating at 45 kDa corresponds to *Tt*HPF protein not fully denatured. These results indicate that *Tt*HPF most likely is a dimer in solution although we cannot rule out other stoichiometries. Probably due to its elongated shape it elutes as a larger protein than the 45 kDa that is expected. Given the nature of HPF, a trimer would be very unlikely. **B)** Micrographs of negative stained ribosome samples. Purified 70S ribosomes serving as control sample shown on left micrograph. Micrograph on the right shows formation of 100S ribosomes after mixing 70S ribosomes with purified *Tt*HPF protein indicating fully active *Tt*HPF protein. Scale bar represents 100 nm. **C)** Chromatogram of fractionated sucrose density gradient showing separation of 100S ribosomes from 70S ribosomes. Material from the 100S ribosome peak was used further in preparation of cryo-grids.

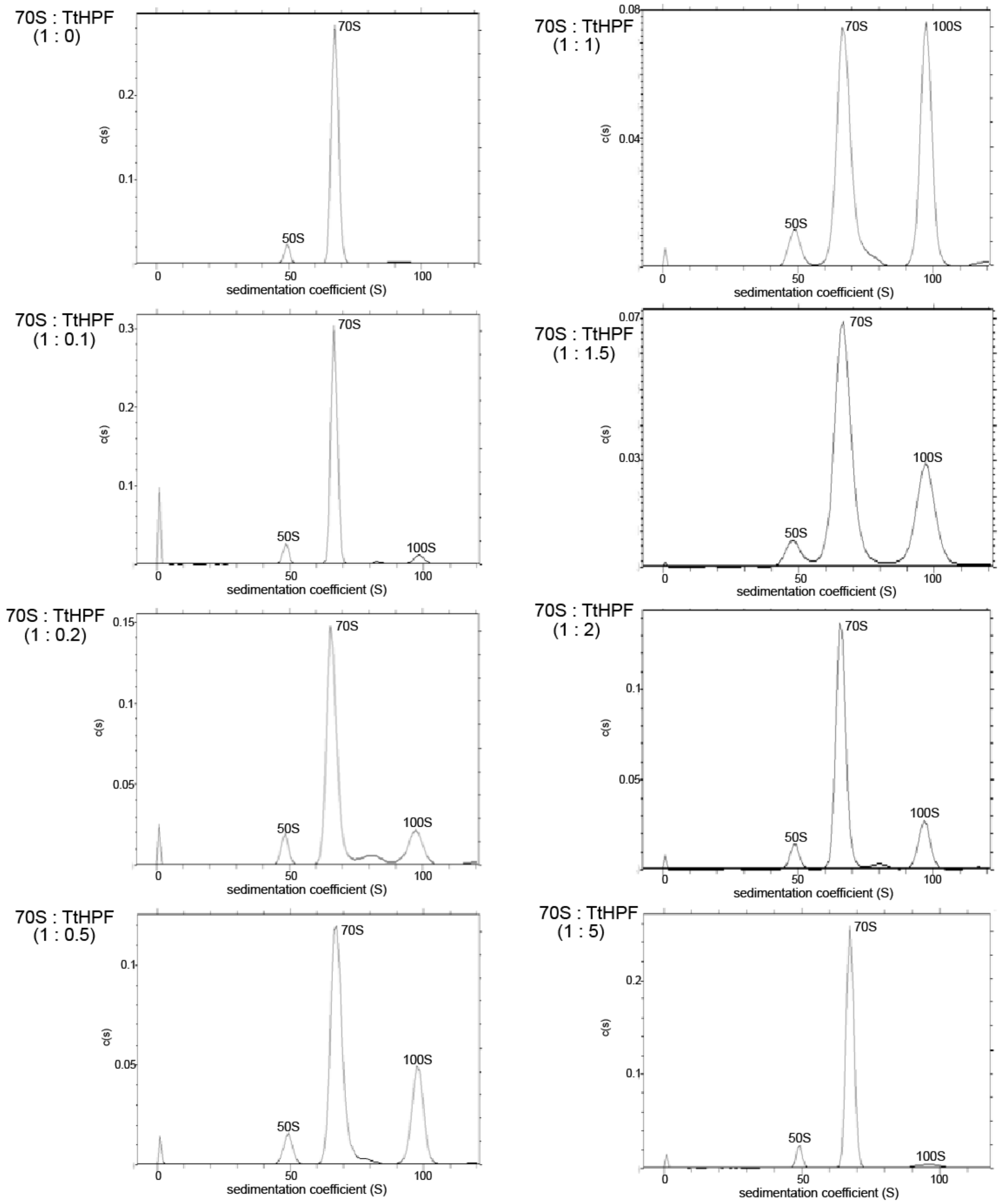

Supplementary Figure 3

Analytical ultracentrifugation results of *in vitro* *Tt*100S ribosome formation dependent of *Tt*HPF molar ratios. The sedimentation profiles show *Tt*70S ribosome as a control followed by seven experiments with 0.1, 0.2, 0.5, 1, 1.5, 2 and 5 times molar ratios of *Tt*HPF. Formation of *Tt*100S ribosome is detected by a 100S sedimentation peak already at 0.1 molar ratio. The formation of *Tt*100S ribosome increases at 0.2, 0.5 and 1 molar ratio where conversion of *Tt*70S ribosome into *Tt*100S ribosome in maximal. At molar ratios of *Tt*HPF at 1.5, 2 and 5 the 100S peak rapidly diminishes. This clearly indicates that formation of *Tt*100S ribosome is dependent on *Tt*HPF protein but at the same time strongly inhibited even at modest molar excess of *Tt*HPF.

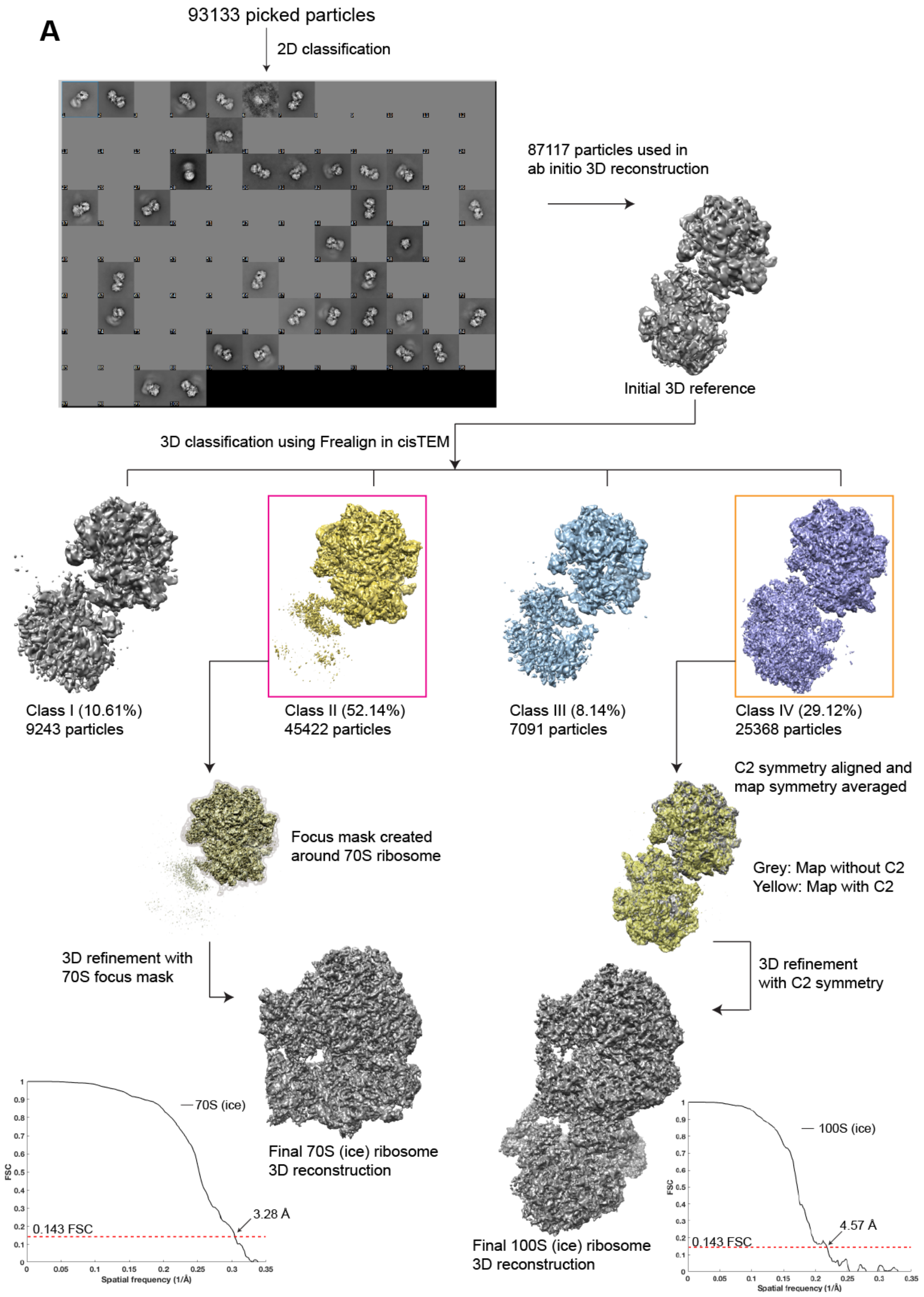
A
93133 picked particles
2D classification
87117 particles used in
ab initio 3D reconstruction
Initial 3D reference
3D classification using Frealign in cisTEM
Class I (10.61%)
9243 particles
Class II (52.14%)
45422 particles
Class III (8.14%)
7091 particles
Class IV (29.12%)
25368 particles
Focus mask created
around 70S ribosome
C2 symmetry aligned and
map symmetry averaged
Grey: Map without C2
Yellow: Map with C2
3D refinement with
70S focus mask
3D refinement
with C2 symmetry
Final 70S (ice) ribosome
3D reconstruction
Final 100S (ice) ribosome
3D reconstruction
70S (ice)
3.28 Å
0.143 FSC
FSC
Spatial frequency (1/Å)
100S (ice)
4.57 Å
0.143 FSC
FSC
Spatial frequency (1/Å)

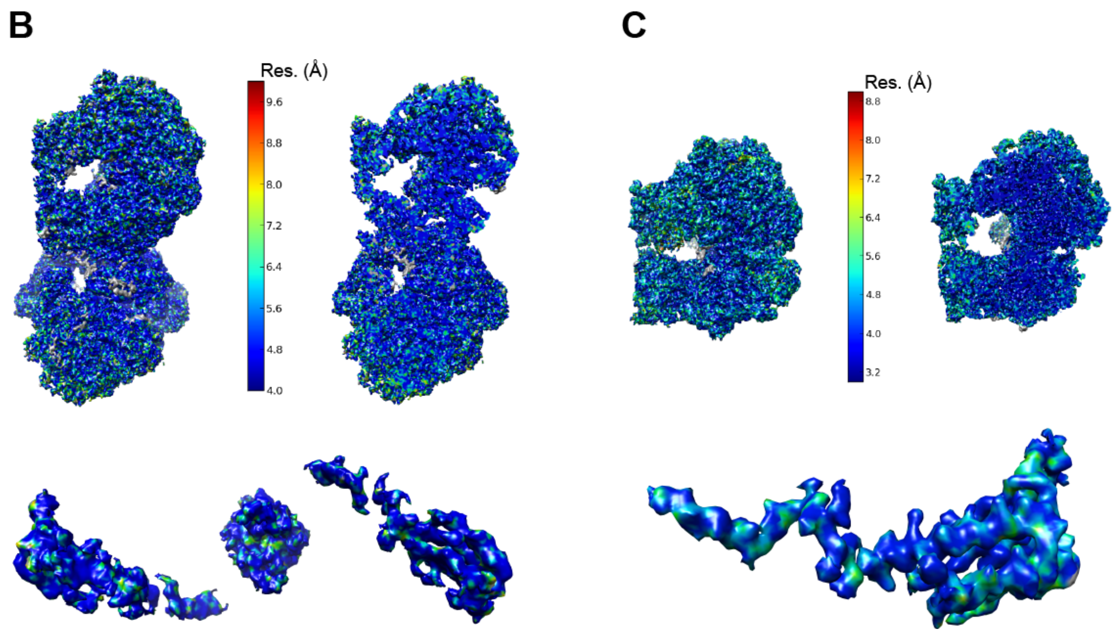

Supplementary Figure 4

A) Overview of single particle processing resulting in final 3D reconstructions 100S (ice) and 70S (ice). Graphs show Fourier shell correlation curves with resolutions at FSC = 0.143 reported as average resolutions of the two maps, respectively. **B and C)** Local resolution map of 100S (ice) in B and 70S (ice) in C. Density maps on left side of local resolution scale bars show full maps whereas on the right side of the scale bars are shown slice-trough of maps. Below the views of the complete densities in B and C, local resolutions of the *Tt*HPF dimer from the 100S (ice) reconstruction (B) and the *Tt*HPF-NTD from the 70S (ice) reconstruction (C) are shown.

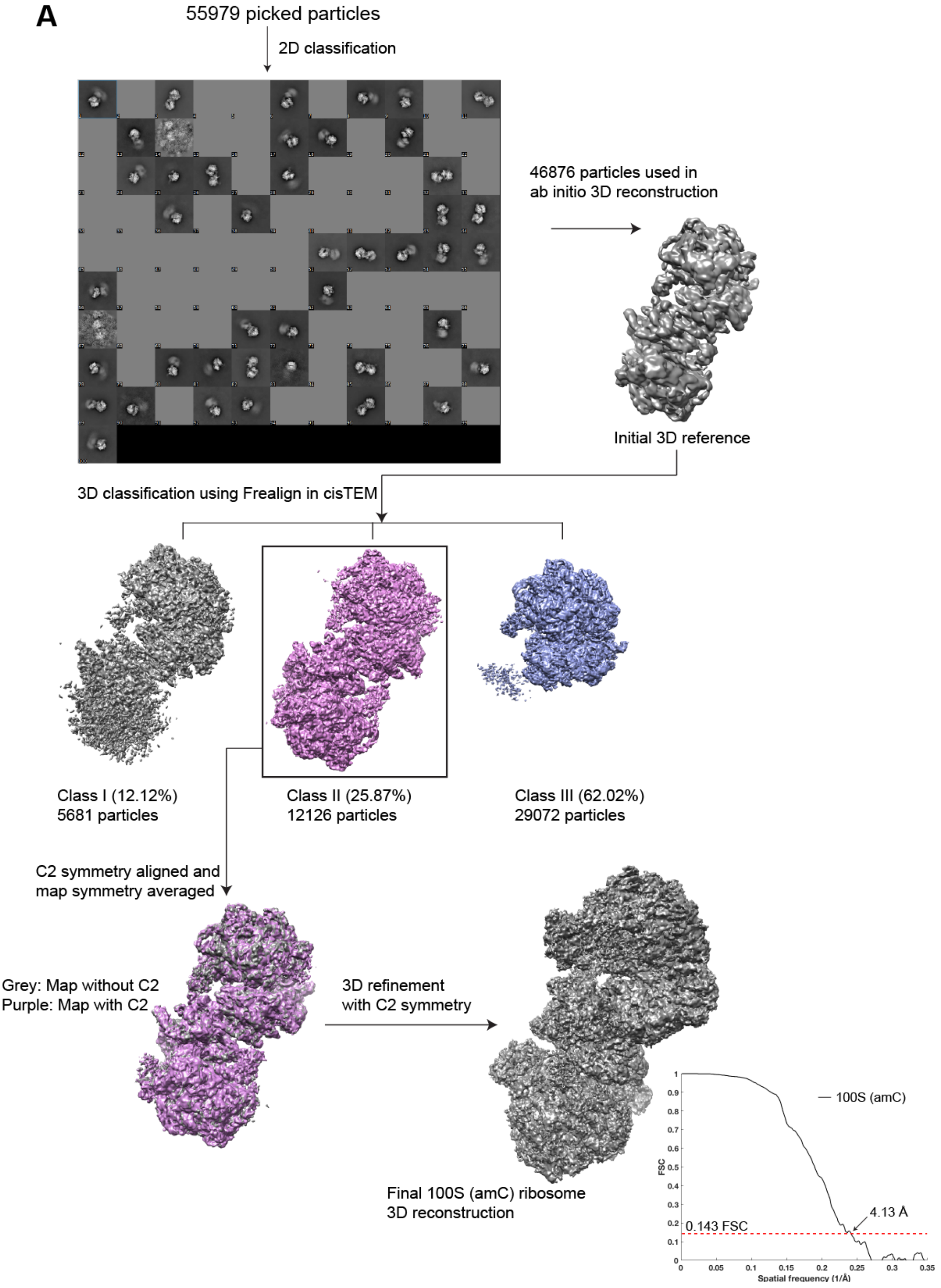
A
55979 picked particles
2D classification
46876 particles used in
ab initio 3D reconstruction
Initial 3D reference
3D classification using Frealign in cisTEM
Class I (12.12%)
5681 particles
Class II (25.87%)
12126 particles
Class III (62.02%)
29072 particles
C2 symmetry aligned and
map symmetry averaged
Grey: Map without C2
Purple: Map with C2
3D refinement
with C2 symmetry
Final 100S (amC) ribosome
3D reconstruction
100S (amC)
4.13 Å
0.143 FSC
FSC
Spatial frequency (1/Å)

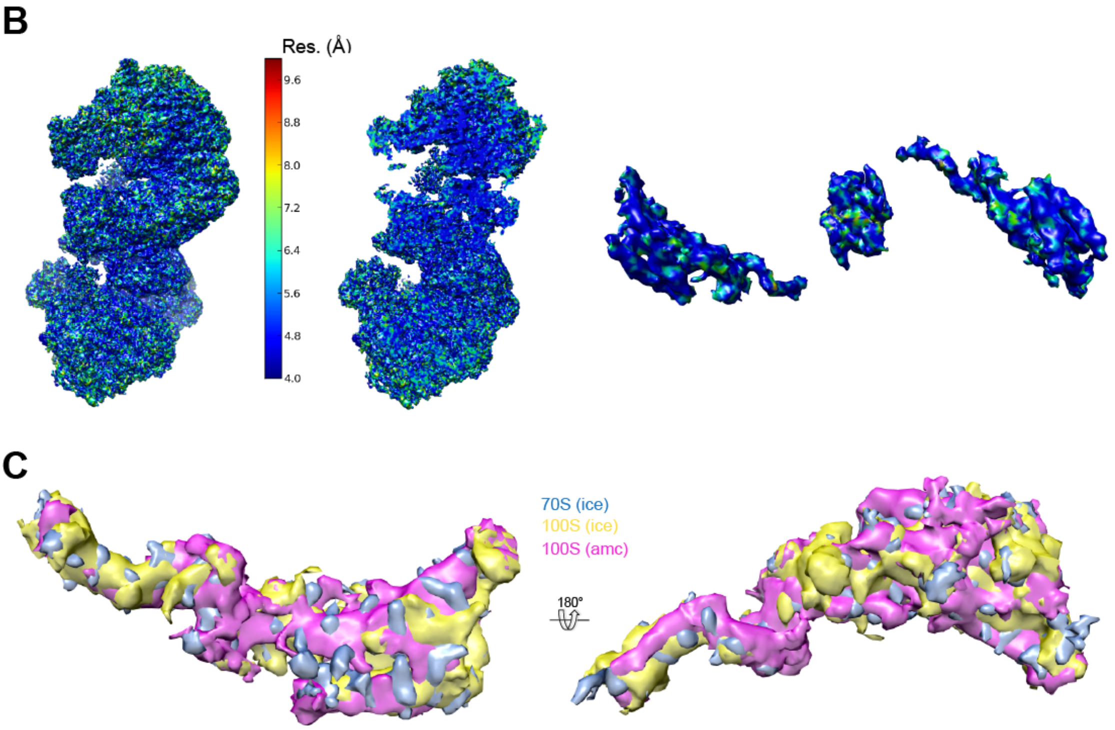

Supplementary Figure 5

A) Overview of single particle processing resulting in final 3D reconstructions 100S (amc). Graph shows Fourier shell correlation curve with resolution at FSC = 0.143 reported as average resolution of the map. **B)** Local resolution map of 100S (amc). Density map on left side of local resolution scale bar shows full map whereas on the right side is shown a slice-trough of the map. To the right of the complete density views, local resolution of the *Tt*HPF dimer from the 100S (amc) reconstruction is shown. **C)** Densities of *Tt*HPF-NTD from the three different reconstructions showing similar conformation of *Tt*HPF-NTD and the linker region.

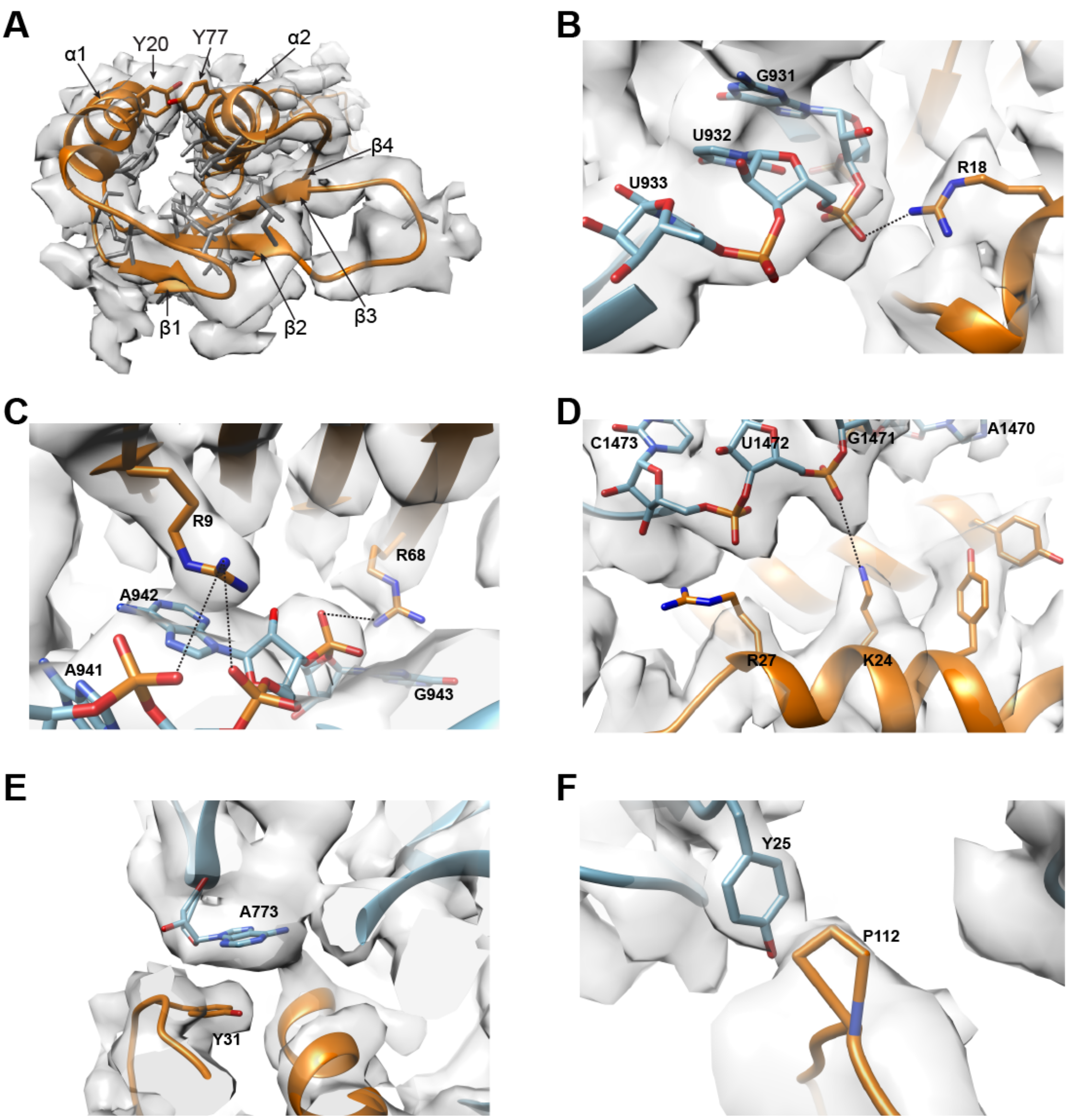

Supplementary Figure 6

Interactions of *Tt*HPF-NTD with the 30S subunit. In all figures *Tt*HPF is colored orange and 30S subunit in light blue. 70S (ice) density is shown as semi-transparent grey surface. **A)** Structure of *Tt*HPF-NTD with hydrophobic residues (colored grey) forming a large hydrophobic core. Tyr20 and Tyr77 from α1 and α2 also form interactions. **B)** *Tt*HPF-NTD Arg18 interacting with phosphate on U932 in h30 of 16S rRNA. **C)** *Tt*HPF-NTD residues Arg9 and Arg68 interacting with phosphate groups of A941, A942 and G943 in loop of h31. **D)** *Tt*HPF-NTD residue Lys24 interacts with phosphate group of U1472 in h44 of 16S rRNA. **E)** Stacking interactions of Tyr31 with A773 in h24 of 16S rRNA. **F)** Residue Tyr25 of ribosomal protein uS11 is within interaction distance of Pro112 in *Tt*HPF linker region.

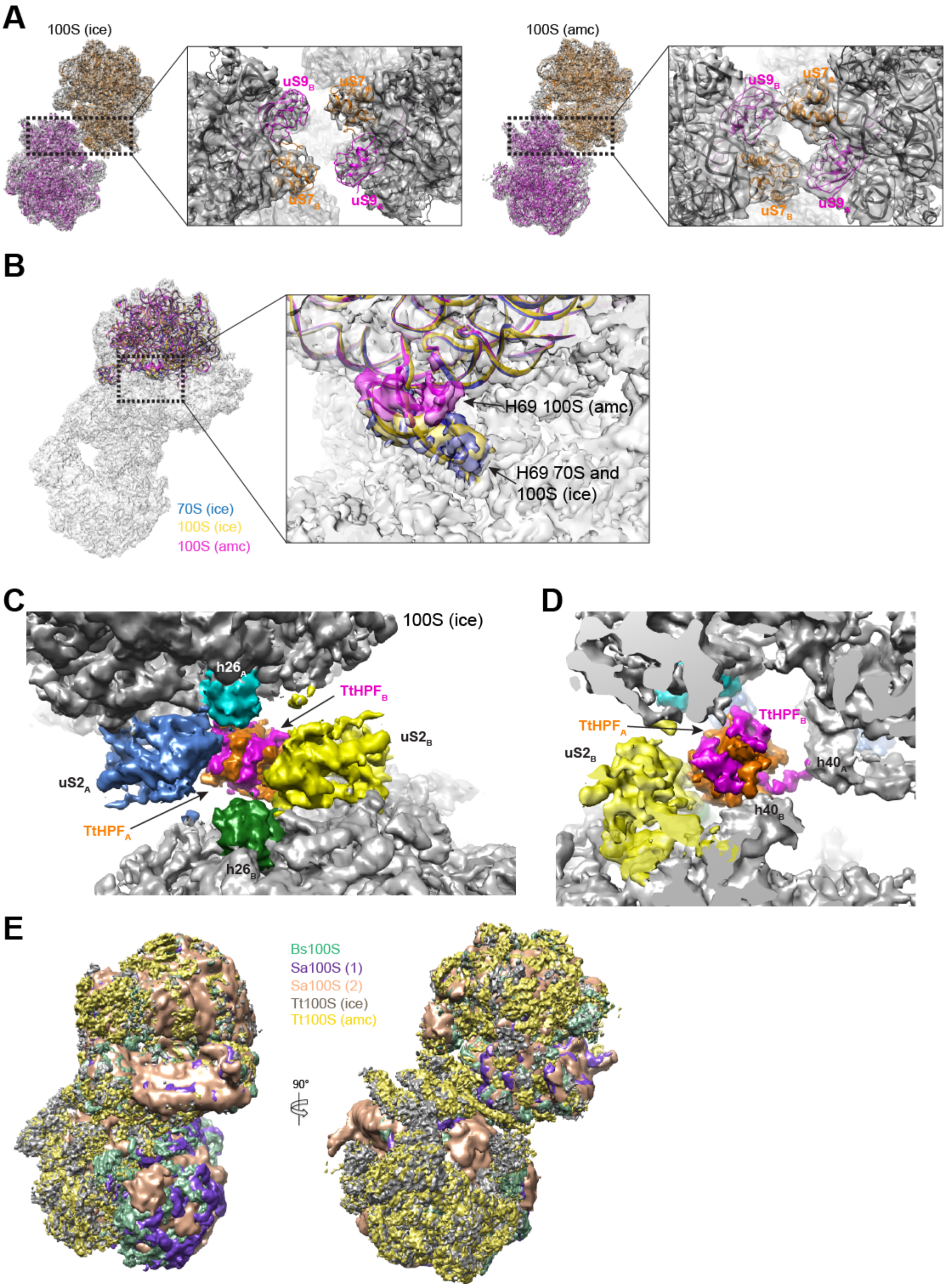
A
100S (ice)
uS9B
uS7A
uS7B
uS9A
100S (amc)
B
H69 100S (amc)
H69 70S and 100S (ice)
70S (ice)
100S (ice)
100S (amc)
C
100S (ice)
h26A
TtHPFB
uS2B
uS2A
TtHPFA
h26B
D
TtHPFA
TtHPFB
uS2B
h40A
h40B
E
Bs100S
Sa100S (1)
Sa100S (2)
Tt100S (ice)
Tt100S (amc)
90°

Supplementary Figure 7

A) 100S (ice) and 100S (amc) EM densities shown as semi-transparent grey surface with ribosome structures fitted (shown in orange and magenta). The close-up views show that uS7 and uS9, located in the back of the head of 30S subunit, do not interact in the 100S (ice) reconstruction whereas in the 100S (amc) reconstruction the two proteins do come into contact. This difference in *Tt*100S ribosome conformation between the two reconstructions could be attributed to genuine conformational difference between the reconstructions, however, it could also be that the difference in 100S (amc) is induced by the amorphous carbon layer used on the grids. **B)** Reconstructions of 70S (ice), 100S (ice) and 100S (amc) aligned and shown with transparent grey surface. 23S rRNA models of the respectively reconstructions are shown in blue for 70S (ice), gold for 100S (ice) and magenta for 100S (amc). Close-up view shows density for H69 of 23S rRNA colored according to respective model. 70S (ice) and 100S (ice) show similar conformations whereas 100S (amc) adopts a very different conformation. Again, this difference in H69 conformation in 100S (amc) could be induced by the cryo-EM experiments using amorphous carbon. **C)** *Tt*100S ribosome dimerization interface in 100S (ice) reconstruction supporting the observations in 100S (amc) that h26 does not interact with uS2*. TtHPF*-CTD chains are colored in orange and magenta, uS2 ribosomal proteins in blue and yellow with h26 colored in cyan and green respectively. **D)** Same colors as in C. As in 100S (amc), 16S rRNA h40 in 100S (ice) as shown here does not interact with *Tt*HPF-CTD homodimer. **E)** Orthogonal views showing superposition of *Tt*100S (ice) and *Tt*100S (amc) reconstructions (grey and gold colored, respectively) with the EM reconstructions of *B. subtilis* 100S (EMD-3664, green) and *S. aureus* 100S (EMD-3637, purple and EMD-3638, salmon). This figure demonstrates that the overall conformation of the 100S is identical although there are subtle differences in the dimerization interface.

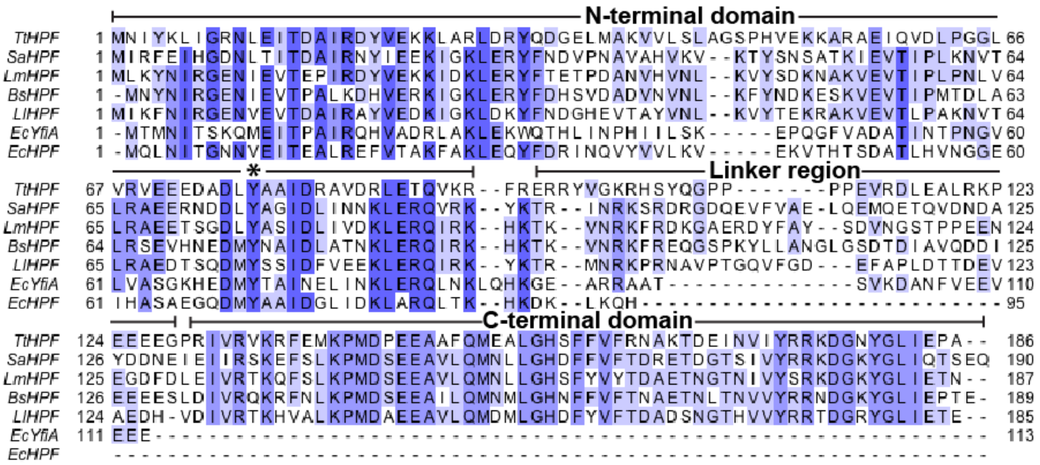

Supplementary Figure 8

Multiple sequence alignment of long HPF protein sequences as well as short HPF and YfiA from *E. coli*. Residues are colored in blue colors based on degree of conservation. The sequence regions corresponding to N-terminal domain, linker region and C-terminal domain are indicated. An asterisk marks the fully conserved Tyr77 residue. The sequences entries are *Tt*HPF (*T. thermophilus* HPF, UniProt entry Q5SIS0 [https://www.uniprot.org/uniprot/Q5SIS0]), *Sa*HPF (*S. aureus* HPF, UniProt entry Q2FIN9 [https://www.uniprot.org/uniprot/Q2FIN9]), *Lm*HPF (*L. monocytogenes* HPF, UniProt entry A0A0H3GEZ8 [https://www.uniprot.org/uniprot/A0A0H3GEZ8]), *Bs*HPF (*B. subtilis* HPF, UniProt entry P28368 [https://www.uniprot.org/uniprot/P28368]), *Ll*HPF (*L. lactis* HPF, UniProt entry A2RIX0 [https://www.uniprot.org/uniprot/A2RIX0]), *Ec*Yfia (*E. coli* YfiA, UniProt entry P0AD49 [https://www.uniprot.org/uniprot/P0AD49]) and *Ec*HPF (*E. coli* HPF, UniProt entry P0AFX0 [https://www.uniprot.org/uniprot/P0AFX0]).

Supplementary Table 1

Cryo-EM data collection, refinement and validation statistics

	70S (ice) / 100S (ice) (EMD-0101 and EMD-0104) (PDB 6GZQ and 6GZX)	100S (amc) (EMD-0105) (PDB 6GZZ)
**Data collection and processing**		
Magnification	59,000x	59,000x
Voltage (kV)	300	300
Electron exposure (e–/Å^2)	35	35
Defocus range (μm)	-0.6 to -4.0	-0.6 to -4.0
Pixel size (Å)	1.1	1.1
Symmetry imposed	C1 / C2	C2
Initial particle images (no.)	93,133	55,979
Final particle images (no.)	45,422 / 25,368	12,126
Map resolution (Å)	3.28 / 4.57	4.13
FSC threshold	0.143	0.143
Map resolution range (Å)	3.0-6.5 / 4.0-7.8	3.9-7.2
CC (model to map fit[a])	0.811 / 0.705	0.715
**Refinement**		
Initial model used (PDB code)	4V9B	4V9B
Map sharpening *B* factor (Å^2)	62.98 / 168.98	121.61
Model composition		
Non-hydrogen atoms	144,138 / 289,188	289,175
Residues	10,360 / 20,830	20,828
R.m.s. deviations		
Bond lengths (Å)	0.017 / 0.010	0.012
Bond angles (°)	1.323 / 1.389	1.547
Validation[b]		
MolProbity score	1.97 / 2.43	2.21
Clashscore[c]	5.52 / 14.61	14.60
Poor rotamers (%)	0.56 / 0.87	1.21
Ramachandran plot		
Favored (%)	84.41 / 78.28	78.53
Allowed (%)	15.12 / 21.58	21.27
Disallowed (%)	0.47 / 0.14	0.20

FSC, Fourier shell correlation; CC, correlation coefficient; RMS, root-mean-square
[a] Across all atoms in model, compiled with Phenix[1]
[b] Compiled using MolProbity[2]
[c] Clashscore; number of serious steric overlaps (>0.4 Å) per 1,000 atoms

Supplementary References

1. Adams, P. D. *et al.* PHENIX: A comprehensive Python-based system for macromolecular structure solution. *Acta Crystallogr. Sect. D Biol. Crystallogr.* **66,** 213–221 (2010).
2. Chen, V. B. *et al.* MolProbity: All-atom structure validation for macromolecular crystallography. *Acta Crystallogr. Sect. D Biol. Crystallogr.* **66,** 12–21 (2010).
